# Pore Engineering in Biomass-Derived Carbon Materials for Enhanced Energy, Catalysis, and Environmental Applications

**DOI:** 10.3390/molecules29215172

**Published:** 2024-10-31

**Authors:** Qi Wang, Bolong Luo, Zhaoyu Wang, Yao Hu, Mingliang Du

**Affiliations:** 1 Key Laboratory of Synthetic and Biological Colloids, Ministry of Education, School of Chemical and Material Engineering, Jiangnan University, Wuxi 214122, China; 17730127768@163.com (Q.W.); 15399706779@163.com (B.L.); 18769411559@163.com (Z.W.); 2 School of Environmental and Ecology, Jiangnan University, Wuxi 214122, China

**Keywords:** biomass-derived carbon, pore engineering, energy, catalysis, environmental

## Abstract

Biomass-derived carbon materials (BDCs) are highly regarded for their renewability, environmental friendliness, and broad potential for application. A significant advantage of these materials lies in the high degree of customization of their physical and chemical properties, especially in terms of pore structure. Pore engineering is a key strategy to enhance the performance of BDCs in critical areas, such as energy storage, catalysis, and environmental remediation. This review focuses on pore engineering, exploring the definition, classification, and adjustment techniques of pore structures, as well as how these factors affect the application performance of BDCs in energy, catalysis, and environmental remediation. Our aim is to provide a solid theoretical foundation and practical guidance for the pore engineering of BDCs to facilitate the rapid transition of these materials from the laboratory to industrial applications.

## 1. Introduction

In recent years, BDCs have attracted significant attention due to their renewability, environmental friendliness, and broad application potential [1,2,3,4]. These materials are produced from renewable resources, such as agricultural waste, forestry by-products, and other organic materials, making them a sustainable alternative to traditional carbon materials, like activated carbon or graphene [5,6]. The renewable nature of BDCs aligns closely with current green technology trends, contributing not only to the reduction of carbon footprints but also to the promotion of a circular economy [7]. Additionally, the diverse range of biomass sources allows for remarkable tunability in BDCs, enabling them to adapt to various application scenarios [8].

One of the key advantages of BDCs lies in their highly customizable physical and chemical properties, particularly in terms of pore structure [9]. Through careful design, BDCs can form complex porous structures with large surface areas, allowing precise control over pore size distribution while optimizing surface chemical properties and functional groups [10]. This tunability significantly enhances their performance in areas such as energy storage, catalysis, and environmental remediation [11,12,13].

Pore engineering is a crucial strategy for significantly enhancing the performance of BDCs across a wide range of applications, particularly in energy storage, catalysis, and environmental remediation [14,15,16,17,18]. The precise control of pore structure, including parameters such as pore size, distribution, and interconnectivity, plays a vital role in determining the effectiveness of BDCs in these fields [19]. A well-tailored pore architecture not only affects the physical properties of the material but also influences its chemical reactivity, surface interactions, and overall functionality [20]. As a result, optimizing pore characteristics can lead to substantial improvements in the efficiency and adaptability of BDCs, making them more suitable for advanced technological applications [21]. By leveraging the unique properties of BDCs and employing pore engineering techniques, these materials can be fine-tuned to meet specific technological needs, further expanding their potential in the fields of energy, catalysis, and environmental protection [14,15,16,22].

There have already been numerous excellent reviews that delve into the properties, sustainability, and environmental benefits of biochar, covering its broad applications in fields such as water treatment, catalysis, soil remediation, energy storage, and construction materials [17,18,23,24,25,26,27,28,29]. However, despite the rapid development of BDCs, existing research often overlooks the critical role of pore engineering in enhancing material performance. Precise control of pore structure is essential for optimizing the performance of BDCs in various applications, and therefore, more systematic and in-depth studies are needed to fully unlock their potential in energy, catalysis, and environmental remediation [30,31,32,33].

**Figure 1:**
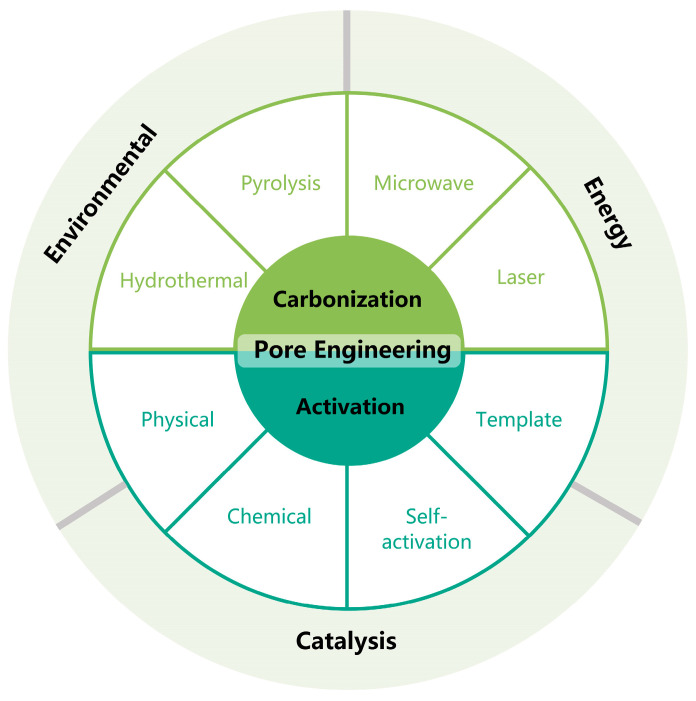
Overview of BDC materials with Pore Structure, Pore Engineering, and wide applications.

As shown in Figure 1, Our review will focus on pore engineering, examining the definition, classification, and regulation methods of pore structures, and exploring their impact on material performance, addressing gaps in current research. The article will systematically introduce various pore regulation techniques, including physical activation, chemical activation, self-activation, and templating methods, with detailed analysis of how these methods are applied in different carbonization processes, such as hydrothermal carbonization, pyrolysis, microwave carbonization, and laser carbonization. Additionally, the review will explore the broad applications of pore structures in energy, catalysis, and environmental remediation, highlighting their potential to enhance material performance.

## 2. Concepts and Classification of Pore Structure

### 2.1. Definition and Classification of Pores

**Figure 2:**
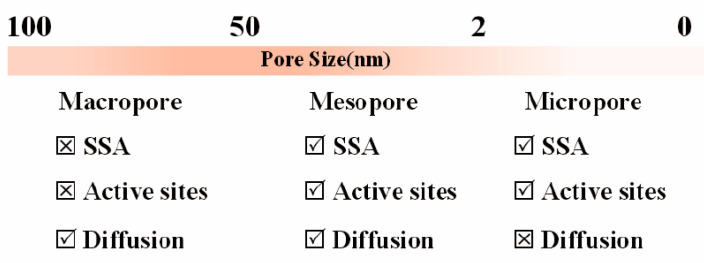
Particle size distribution and characteristics of various types of pores.

Owing to the unique properties of BDCs, the design and construction of their porous structures in materials are of great significance during their preparation process [34]. As shown in the Figure 2, pores in materials can be categorized into three types: micropores (pore size less than 2 nm), mesopores (pore size of 2–50 nm) and macropores (pore size more than 50 nm). Microporous materials are renowned for their extremely high surface areas and a pore structure composed of minuscule channels and cavities, endowing the materials with unique interactive capabilities at the molecular level. Mesoporous materials, on the other hand, feature an orderly pore structure along with relatively larger diameters, offering considerable flexibility in pore size adjustment; their pore walls may be continuous or discontinuous, and sometimes they include regularly arranged channels. Macroporous materials are distinguished by their spacious pore structures and larger diameters, often manifesting a more open pore network that permits the free movement of large molecules or particles; thus, macroporous materials typically exhibit greater permeability.

### 2.2. Impact of Pore Structure on Performance

Micropores play a crucial role in increasing the specific surface area of materials, as they provide abundant inner surface areas. When it comes to further increasing the specific surface area, mesopores play a positive role, not only increasing the additional surface area, but also promoting the transport and diffusion of gas molecules within the material. As for macropores, their main function is to affect the macroscopic structure and pore network of materials, and they play a crucial role in improving the quality transfer efficiency of materials [35,36]. Overall, the synergistic effect of micropores, mesopores, and macropores determines the specific surface area and pore characteristics of materials, thereby affecting their performance in various applications. In catalytic reactions, the high surface area of microporous materials provides numerous active sites for catalytic reactions and gas adsorption [37,38], which are specific regions on the catalyst surface that can interact with reactants and participate in the reaction. The active site plays a crucial role in reducing the activation energy and accelerating the reaction rate [39]. However, the small pore size of micropores may limit the diffusion path of substances, affecting their efficiency in applications that require rapid material transport [40]. In contrast, although mesoporous and macroporous materials may have slightly inferior surface area, their larger pore size and ordered pore structure provide advantages for the accessibility of active sites and material transport, which is particularly important in heterogeneous catalytic reactions. In the field of energy storage, the porosity and pore size distribution of porous carbon materials as electrodes for supercapacitors play a crucial role in ion adsorption and transport rates [41,42]. Micropores help increase the surface area and storage capacity but may sacrifice transmission rate. Meanwhile, mesopores and macropores can enhance the ion transfer rate, thereby increasing the power density of supercapacitors [42]. Environmental management also benefits from the pore size and structure of porous materials, which determine their ability to adsorb pollutants [43]. Microporous materials perform better in adsorbing small molecule pollutants, while mesoporous and macroporous materials are more suitable for capturing large molecule or particle pollutants [41,44]. Therefore, the pore structure of porous materials not only determines their surface area and distribution of active sites, but also directly affects the efficiency of material transport and application related properties.

## 3. Main Methods for Regulating Pore Structure

### 3.1. Physical and Chemical Activation

Activation is a process during which different kinds of gas or activators can be used to act with carbonaceous materials [45]. The activation process comprises two steps: carbonization and activation. In the carbonization stage, biomass undergoes carbonization under high temperature and anaerobic conditions to form carbon materials. At the same time, non-carbon elements are expelled in the form of gas, resulting in the formation of pores [46]. Activation is a crucial step in controlling the morphology of carbon materials, and hierarchical porosity is continuously introduced on the surface of the carbon material in this process, which increases its specific surface areas (SSAs), enriches its pore structure, and beneficially improves the overall physicochemical properties of the carbon material. Activation is a key step in controlling the morphology of carbon materials. Hence, it is extremely important to design activation process parameters reasonably (e.g., activation temperature and the activator) [47]. Activation can be divided into four types: physical activation, chemical activation, self-activation and the template method.

In the physical activation process, biomass will go through two stages. The first stage is the pyrolysis of biomass precursors in an inert gas atmosphere, which will lead to partial carbonization and the elimination of non-carbon elements. The second stage is activation of the carbonized material with an oxidizing gas at elevated temperatures (800–1100 °C) to adjust the pore structure, with the entire activation process taking place in an oxidizing gas medium [48].

**Figure 3:**
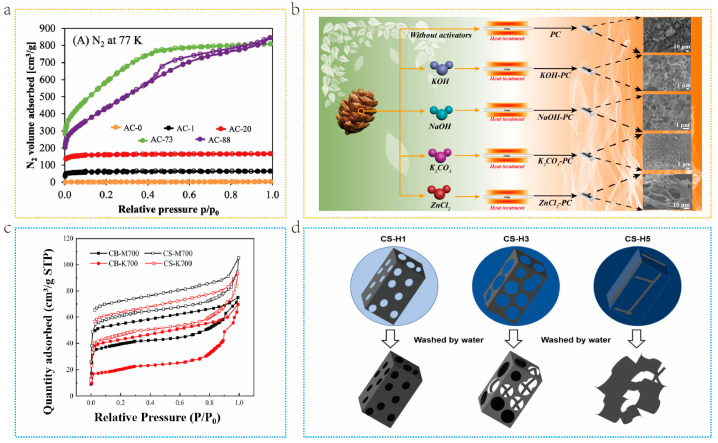
(**a**) Adsorption/desorption isotherms of nitrogen at 77 K (the AC-0 sample was heated under an inert atmosphere at 1273 K. All the other samples were physically activated with carbon dioxide at 1273 K. Burn-offs of 1%, 20%, 73%, and 88% were confirmed for materials activated for of 15, 60, 120 and 240 min, respectively. Samples were named according to measured burn-off, i.e., AC-1, AC-20, AC-73, and AC–88, respectively.) [49]. Copyright 2022, Elsevier. (**b**) Adsorption/desorption isotherms of different heating rates [50]. Copyright 2022, Elsevier. (**c**) Schematic illustration of the preparation of porous carbonaceous treated by different activators [58]. Copyright 2020, Elsevier. (**d**) Carbon formation with different amounts of KOH [59]. Copyright 2020, Elsevier.

Activation time is a crucial parameter in physical activation. Zgrzebnicki et al. [49]. prepared N-doped activated carbon derived from furfuryl alcohol through CO_2_ activation. Different activation times of 15, 60, 120, and 240 min were set. The variation in the pore structure of the material with activation time is presented in Figure 3a. Apparently, the first three materials can all be classified as microporous materials. The latter two materials are porous materials with the coexistence of micropores and mesopores. Carbonization heating rates are also an important factor. Jing et al. [50]. reported a simple and scalable strategy for preparing activated carbon, aiming at producing activated carbon with a developed pore structure in high yield through rapid pyrolysis physical activation. Compared with slow pyrolysis semi-coke (M) of the same particle size, fast pyrolysis semi-coke (K) has a lower nitrogen adsorption capacity, indicating that a high heating rate is not conducive to the formation of micropores. The “hysteresis loop” of K shows a higher degree of separation, indicating that high heating rates promote the formation of mesopores and macropores (Figure 3b). Physical activation is an effective technique for improving the specific surface area and porosity of biomass-derived carbon materials, which is crucial for enhancing their performance in adsorption [51], catalysis, and energy storage [52,53]. This method is environmentally friendly, especially when using water vapor or carbon dioxide as activators, which can reduce the use of chemical additives [54]. However, precise regulation of pore structure to meet specific application requirements still faces challenges. In addition, the high temperature and prolonged processing during the physical activation process may result in high energy consumption, which to some extent limits its application on an industrial scale [55]. Future research and development work needs to focus on optimizing activation conditions to achieve improved production efficiency and material properties, while maintaining environmental sustainability.

Unlike physical activation, in chemical activation, activators are added. Here, carbonization and activation take place simultaneously in a single step [56]. During activation, the activator and precursor become involved in dehydration, cross-linking, or condensation reactions, with carbon atoms etched and separated from the carbon skeleton to form a porous structure. In addition, diffusion of the generated gas molecules (CO_2_, CO, and H_2_O) leads to the formation of many pores. Common chemical activators are currently divided into three categories: alkaline (e.g., KOH and NaOH), acidic (e.g., H_3_PO_4_ and H_2_SO_4_), and molten salt (e.g., ZnCl_2_, Na_2_CO_3_, and K_2_CO_3_) [57].

Treating pinecones with different chemical activators could yield porous carbon (PC) of different morphologies (Figure 3c). The presence of KOH and ZnCl_2_ produces micropores, which are different from those of NaOH and K_2_CO_3_ activated porous carbon. Among them, the sample activated by K_2_CO_3_ has an ordered, dense, and honeycomb-like porous structure [58]. The amount of activator used is also crucial. Yang et al. [59] prepared porous carbon CS-H3 using dry wood sawdust and KOH as an activator. The pore size distribution of carbon microstructure was regulated by the content of KOH and the addition of heteroatoms, as in the illustration for Figure 3d. At a lower KOH content in MS (CS-H1), the limited oxidizing action of KOH limits the compatibility of MS and carbon, and thus reduces the template action of MS to form pores. The MS deeply penetrates the carbon phase with a larger amount of KOH used (CS-H3 and CS-H5), making the formation of an open pore structure and even sheets. Compared with chemical activation, physical activation demands a longer activation time, a higher activation temperature, and consumes a substantial amount of energy [60]. Chemical activation is more effective than physical activation in improving the specific surface area and pore structure of biomass-derived carbon materials, especially in the generation of micropores [61]. By precisely adjusting the preparation conditions, chemical activation can customize the control of pore size and pore distribution to meet diverse application needs. Another major advantage of this method is its wide applicability to raw materials, which can convert biomass materials, such as agricultural waste and forestry by-products, into high-value porous carbon materials, achieving resource recycling [62]. Although chemical activators, such as KOH, have relatively low costs, some activators may be expensive and may have potential negative impacts on the environment, especially those that are difficult to recycle and may increase environmental burdens [54]. In addition, chemical activation requires precise control of reaction conditions, which increases the complexity of the operation and places higher demands on the accuracy of the production process. Therefore, future research and development should focus on reducing the cost of chemical activation, minimizing environmental impact, while improving material performance and production efficiency to achieve more sustainable and economically efficient production processes.

### 3.2. Self-Activation

**Figure 4:**
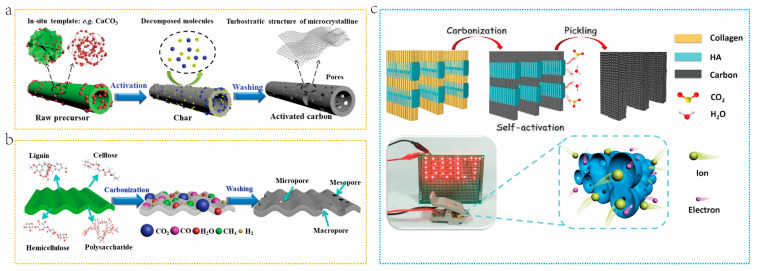
(**a**) Activation mechanism of self-activating agent via in situ template [54]. Copyright 2020, Elsevier. (**b**) Activation mechanism of self-activating agent via pyrolysis gases [54]. Copyright 2020, Elsevier. (**c**) Preparation of mesoporous dominant porous carbon by direct carbonization of bovine bone [66]. Copyright 2017, American Chemical Society.

Self-activation is a strategy that does not rely on the addition of external activators. Its principle is that biomass precursors are abundant in inherent inorganic compounds, which can activate them in situ and convert the precursors into porous carbon [63]. Currently, some studies have reported the utilization of organic acid salts of Na, K, and Ca as carbon precursors and self-activators [64]. The principle is shown in Figure 4a. Certain metal nanoparticles and corresponding oxides are generated during the pyrolysis process. These can act as in situ templates and can be removed by thermal or etching methods. Subsequently, the originally occupied area is released and transformed into pores, thus achieving permeation into the carbon matrix at the molecular level to regulate the pore structure. Biomass is typically composed of lignin, cellulose, hemicellulose, or polysaccharides. These components produce H_2_, CO_2_, CO, H_2_O, and CH_4_ during the activation stage [65]. Therefore, these pyrolysis gases can be used as self-activators. The potential activation mechanism is shown in Figure 4b. During the activation process, these gases can laterally transfer or vertically penetrate carbon nanosheets, leading to the formation of pores. Jing et al. [66]. prepared porous carbon (PC) with high defect density by directly carbonizing bovine bone containing hydroxyapatite (HA), without the need for any additional activators or templates. Through in situ self-activation of HA, PC with a hierarchical porous structure dominated by mesopores was obtained (Figure 4c). Self-activation technology stands out in the field of BDC preparation due to its environmental and economic benefits. This technology not only reduces the generation of chemical waste by omitting additional chemical activators [61], but also simplifies the traditional two-step activation process by combining carbonization and activation processes into one step, effectively reducing production costs. However, the unevenness of gas distribution during self-activation may lead to inconsistencies in the pore structure of carbon materials. In addition, compared to BDCs prepared using chemical activators, BDCs obtained by self-activation methods usually have smaller SSAs and pore size [54]. With advancement in technology, these challenges may gradually be overcome, making self-activation a mainstream method of biomass carbonization.

### 3.3. Templating Methods

During this period, the template is impregnated into the biomass precursor. After carbonization and removal of the template with strong acid or alkali, uniformly sized pores will be generated [67]. The most prominent feature of this method is that the type, structure, spatial size, and shape of the template can control and adjust the structure of the material [68].

**Figure 5:**
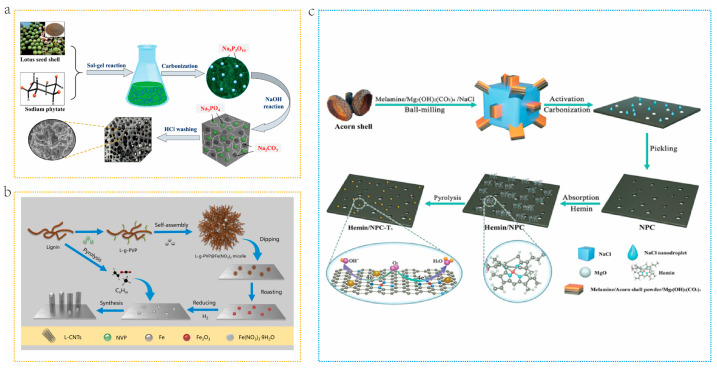
(**a**) Schematic illustration of the preparation of natural biomass-derived hierarchical porous carbons [71]. Copyright 2023, Elsevier. (**b**) Preparation of L-CNTs [72]. Copyright 2018, American Chemical Society. (**c**) Schematic illustration of the proposed procedure for fabricating Hemin/NPC catalysts [80]. Copyright 2021, Wiley Online Library.

The hard template method, also known as the “nano casting method”, makes use of the excellent spatial control offered by the template to achieve ordered micro-size and three-dimensional structure arrangement in carbon materials [67]. Typical hard templates include MgO, ZnO, and SiO_2_ [69]. The main steps of the hard template method are as follows: (1) Select or synthesize the required materials as the hard template. (2) Thoroughly and effectively mix carbon sources and hard templates. (3) Conduct pyrolysis under certain conditions at high temperatures. 4) Rinse and remove the hard template with chemical reagents [70]. Hu et al. [71] took lotus seed shells as carbon precursors and sodium phytate as hard template precursors to prepare natural biomass-derived porous carbon with a hierarchical porous structure through in situ template combined with NaOH activation. As shown in Figure 5a, the lotus seed shell and sodium phytate aqueous solution are fully mixed at 60 °C to form a mixed gel. During carbonization, soluble sodium phytate undergoes pyrolysis to form nano Na_5_P_3_O_10_, which then reacts with NaOH to transform into nano Na_2_CO_3_ and nano Na_3_PO_4_ particles. These particles are uniformly dispersed in the carbon substrate and leave large mesopores/macropores after washing treatment. A well-developed hierarchical porous carbon with a hollow nest structure is obtained by combining the micropores generated by NaOH activation. Compared with the hard template method, the soft template method does not require the use of a corrosive agent to removal the template, which improves synthesis efficiency [72].The soft template method employs organic compounds as templates, including surfactants, block copolymers, and ionic micelle [73]. Selecting appropriate carbon precursors and template materials is crucial for ensuring the use of soft templates for directional synthesis. The soft template method typically involves ordered aggregates formed by amphiphilic molecules, such as block copolymers and surfactants. When the concentration exceeds the critical concentration, these molecules will self-assemble into micelles or vesicles and interact with carbon precursors through hydrogen bonding, hydrophobic or hydrophilic interactions, and electrostatic interactions to form coatings on the precursors [74]. During the subsequent carbonization process, the micelles decompose to form a unique pore structure [75]. Tu et al. [72] prepared catalyst CNTs from lignin by employing a soft template method (Figure 5b). They controlled the size and dispersion of catalyst particles by loading the catalyst onto nano micelles. The hard template method has shown excellent performance in preparing highly ordered porous carbon materials, which can precisely control the pore size and pore shape. However, this advantage comes with the high cost and environmental risks of using strong acids or bases to remove the template [76]. Compared with this, the soft template method is favored for its simple operation process and environmental friendliness, and the template is easy to remove during high-temperature carbonization [77]. However, the soft template method may require more expensive template agents during the synthesis process and, due to insufficient filling of raw materials, it may lead to discontinuities and structural defects in the product pores [78]. In addition, mesoporous carbon materials prepared by this method typically have lower surface area and pore volume [79]. Therefore, to combine the advantages of both, a dual template method was introduced. Lu et al. [80]. synthesized FeOx embedded Fe-N doped carbon (Hemin/NPC) via carbonization by employing acorn shell and Mg_5_(OH)_2_(CO_3_)_4_ and NaCl as dual templates. The removal of template by acid washing generates abundant mesopores, while the melted NaCl nano-droplets were immersed into carbon framework to produce porous structure (Figure 5c).

## 4. Impact of Carbonization Methods on Pore Structure

The above activation methods can regulate the structure of BDC. However, high-temperature carbonization is still required to convert biomass into BCM. This process mainly includes decomposition, polymerization, aromatization, carbonization, and graphitization. At the same time, different carbonization methods also affect the structure of BDC, such as hydrothermal carbonization (HTC), pyrolysis, and other methods.

### 4.1. Hydrothermal Carbonization

Hydrothermal carbonization (HTC) is a green method for preparing carbon containing materials using biomass, such as cellulose, lignin, and hemicellulose, as raw materials. It is usually carried out in a temperature range of 100–250 °C and a pressure range of 2–6 MPa, with an aqueous solution as the medium [81]. During the HTC process, as the temperature increases, the water vapor pressure increases sharply, reducing surface tension. By simulating the natural coalification process, carbon materials with unique structures are produced [75]. Several reactions occur during the HTC process, including condensation, polymerization, hydrolysis, decarboxylation, dehydration, and aromatization.

**Figure 6:**
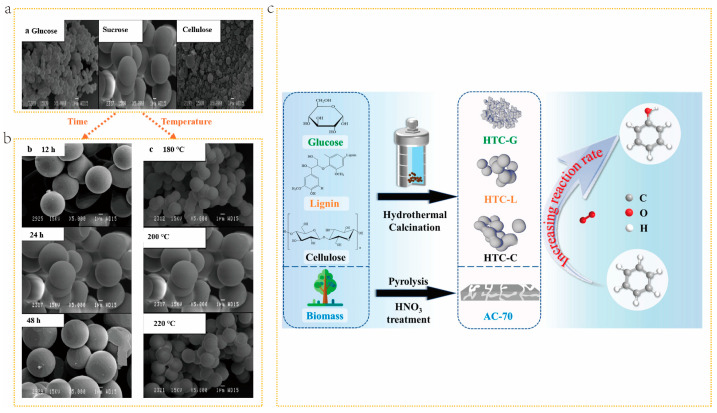
(**a**) The influence of biomass type [82]. Copyright 2014, Elsevier. (**b**) The influence of temperature and reaction time [82]. Copyright 2014, Elsevier. (**c**) Preparation of BDCs from different types of biomass through hydrothermal carbonization [84]. Copyright 2021, American Chemical Society.

The preparation conditions of the HTC method, such as HTC temperature, residence time, catalyst usage, and water-to-biomass ratio, have a significant impact on the structure of the obtained BDCs. Romero et al. [82] prepared carbon spheres by using sucrose, glucose, and cellulose as carbon sources via the hydrothermal method. As shown in Figure 6a after hydrothermal treatment, all three types of biomasses formed spherical carbon structures under the same preparation conditions. However, the diameter of the carbon spherical varied with changes in carbon source. At the same time, the temperature and reaction time of hydrothermal carbonization can lead to differences in the diameter of carbon spheres. Taking sucrose spherical carbon as an example, as the temperature increases, the spherical structure starts to develop from 180 °C, and the maximum size of spherical carbon appears at 200 °C. As the temperature continues to rise, the size of the sphere begins to decrease, which can be ascribed to the complete decomposition of the raw materials (Figure 6b). Regarding the HTC residence time, it can be observed that, after 12 h, spherical carbon is not completely formed. After a certain period, spherical carbon with a definite morphology is obtained (Figure 6b). Additionally, the continuous increase in time has little effect on the structure of spherical carbon. Residence time will also affect the porosity of BDCs, and longer residence time is beneficial to the formation of defined structure porosity, pore volume, and high SSAs. Due to the different thermal decomposition behaviors of biomass components, different biomass precursors will have an impact on the structure of BDCs prepared by the HTC method. For example, BDCs with a higher lignin content produce more biochar than those rich in hemicellulose or cellulose, as lignin degradation is difficult [83]. Chen et al. [84]. prepared hydrothermal carbon from glucose, lignin, and cellulose. As shown in Figure 6c, for HTC-G (glucose), its spherical primary particle size is approximately 200 nm and the surface is smooth. HTC-L (lignin) and HTC-C (cellulose) also display similar primary particle shapes, but with average sizes of 4.2 and 6.0 µm respectively. Additionally, the activated carbon AC-70 obtained by direct high-temperature pyrolysis of glucose shows irregular block-shaped particles with a size of over ten microns. This once again demonstrates that hydrothermal treatment is more conducive to the formation of spherical BDCs compared to direct pyrolysis. The mild temperature of the hydrothermal method helps to cultivate crystals with almost no defects and good orientation [85]. In addition, the hydrothermal method is highly favored for its green and environmentally friendly characteristics. It can not only synthesize small single crystals of a single component, but also prepare various complex compound powders, including composite oxides [86]. However, the demand for high-temperature and high-pressure environments in hydrothermal methods has increased their dependence on specific production equipment. The expansion of fluid volume in a closed reaction vessel poses safety hazards, which to some extent limit the widespread application of hydrothermal methods [87]. To promote the further development of hydrothermal methods, future research should focus on developing safer and more efficient synthesis technologies, reducing dependence on equipment, and fully leveraging its unique advantages in the field of material synthesis.

### 4.2. Pyrolysis Carbonization

Pyrolysis carbonization is another practical approach for preparing BDCs. It entails the heating and decomposing of organic biomass under an inert gas atmosphere. In this process, volatile substances and non-carbon elements are eliminated, leading to the decomposition of biomass into solid carbon and gaseous products. The structure and porosity of pores formed within carbon materials can be effectively adjusted by controlling carbonization parameters, like heating rate, carbonization temperature, and annealing time [88].

**Figure 7:**
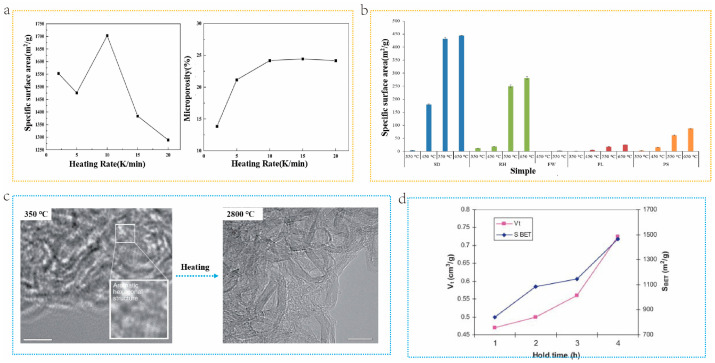
(**a**) The influence of heating rate on SSA and on porosity [90]. Copyright 2020, Elsevier. (**b**) The influence of carbonization temperature on SSA [91]. Copyright 2020, Elsevier. (**c**) SEM images of ilk protein at different temperatures [93]. Copyright 2015, Nature. (**d**) The effect of insulation time on SSA and pore volume [94]. Copyright 2012, Elsevier.

Heating rates are one of the crucial operating parameters that exert a significant influence on the structure of BDCs. Research indicates that rapid heating facilitates the formation of three-dimensional structures in carbon materials. Conversely, slow heating results in high porosity and high SSAs [89,90]. Zhang et al. [90]. prepared porous carbon materials by using the macromolecular components of biomass pyrolysis steam and calcium citrate as carbon sources and templates, respectively. They also investigated the effect of heating rate on the structure of BDCs. As depicted in Figure 7a, with acceleration in the heating rate, the specific surface area and micropore volume of porous carbon initially increase and then decrease, reaching their peak at 10 K/min. When the heating rate increases, the pyrolysis vapor and calcium citrate can be fully mixed, which is beneficial for the formation of a pore structure dominated by mesopores and micropores in the subsequent carbonization process. However, when the heating rate is overly fast, excessive steam is generated and the amount of calcium citrate is insufficient, leading to the collapse of the pore structure and a significant reduction in SSAs. The carbonization temperature is another crucial preparation condition that impacts the carbon structure. Pariyar et al. [91] compared biomass materials obtained from five raw materials, pine sawdust (PD), rice husk (RH), food waste (FW), poultry waste (PL), and papermaking sludge (PS), at different pyrolysis temperatures (350, 450, 550, and 650 °C) and found that the pyrolysis temperature also affects the SSAs of BDCs. As shown in Figure 7b, the SSAs of the five types of BDCs increase with the rise in carbonization temperature. On the other hand, the degree of graphitization of carbon materials is influenced by the carbonization temperature. At a lower temperature (such as 600 °C), the graphitization degree of BDCs is relatively low. However, a high degree of graphitization can be attained at a high carbonization temperature (such as 1100 °C) [92]. Cho et al. [93]. systematically studied the structural and chemical changes of silk protein at temperatures above the onset of thermal degradation, using silk protein as a carbon source. Silk protein undergoes a structural transformation into a sp^2^ hybridized carbon hexagonal structure by simple heating to 350 °C. When heated to 2800 °C degrees Celsius, a highly developed graphite structure is formed, as shown in Figure 7c.

During the pyrolysis process, holding time is extremely important. Bouchelta et al. [94]. investigated the effect of pyrolysis conditions (holding time) on the preparation of BDCs from jujube seeds. As shown in Figure 7d, holding time has a significant impact on the comparison of surface area and pore volume. Increasing the holding time is beneficial for improving pore volume and SSAs. This is because the longer the holding time, the greater the mass of volatile compounds released, resulting in higher SSAs and porous volume. In addition, studies have shown that longer insulation times lead to BDCs having fewer heteroatoms and a higher degree of graphitization [95]. The pyrolysis method is carried out under moderately low temperature and an inert atmosphere, which is conducive to finely controlling the graphitization degree and surface functional groups of carbon materials, thereby precisely adjusting their physical and chemical properties [96]. However, the carbon materials prepared by this method may face the problem of insufficient carbonization degree. In addition, uneven heating during pyrolysis may lead to inconsistent quality and performance of carbon materials [97]. Meanwhile, potential by-products generated during the process, such as tar and gas, need to be effectively treated to prevent environmental pollution [96]. Therefore, although pyrolysis has shown great potential in converting biomass into carbon materials, further optimization of reaction conditions and post-treatment steps is still needed to enhance the performance of carbon materials and ensure their environmental sustainability.

### 4.3. Microwave and Laser Carbonization

**Figure 8:**
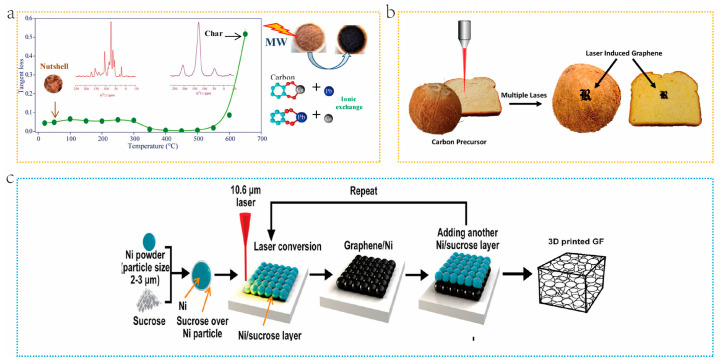
(**a**) Microwave pyrolysis of biomass [100]. Copyright 2017, Elsevier. (**b**) Schematic diagram of selecting specific areas of biomass to produce conductive graphite carbon [103]. Copyright 2017, American Chemical Society. (**c**) Schematic of in situ synthesis of 3D GF using a simulated 3D printing process [103]. Copyright 2017, American Chemical Society.

Microwave carbonization (MAP) is a novel carbonization approach. The MAP mechanism involves converting electromagnetic energy into heat within the irradiated material [98]. MAP employs electromagnetic waves (EMWs), having wavelengths ranging from 1 mm to 1 m and frequencies ranging from 300 MHz to 300 GHz. In comparison with traditional techniques, MAP is a rapid, efficient, and comprehensive process of heating from within the material and has high safety. Owing to the swift carbonization process of MAP, it is difficult to control heating by adjusting the heating rate. The irradiation time and energy density are controllable parameters. Microwaves can open the closed pores in carbon materials during the carbonization process, thereby increasing their specific surface area. Nevertheless, the MAP time cannot be prolonged indefinitely [99]. Jimenez et al. [100] reported on BDC through microwave carbonization of walnut shells. They observed that, when the energy absorbed by the material surpasses a certain value, the micropores on the material surface will collapse due to excessive microwave heating, leading to decreased SSAs (Figure 8a). MAP has advantages of high heating efficiency, easy operation, and environmental friendliness in the preparation of biomass derived carbon materials [101], but there are also challenges, such as uneven energy absorption and high equipment costs. For example, when the microwave energy absorbed by a material exceeds a certain value, it may lead to a loss of specific surface area of the material [102], as further microwave heating may cause the collapse of micropores.

Laser-induced graphitization (LIG) is a method for converting biomass into carbon materials. Lasers can generate extremely high temperatures in a short time, enabling them to be used to induce the carbonization of biomass. The most prominent feature of this method is that it can select specific regions of biomass to produce conductive graphite carbon, as shown in Figure 8b. Additionally, metal catalysts and gases are not required. Tour et al. [103] prepared freestanding three-dimensional graphene foam (GF) by laser writing a Ni/sucrose mixture in a hydrogen atmosphere (as shown in Figure 8c). During the laser-induced carbonization (LIC) process, nickel powder and sucrose can absorb laser energy and be heated to high temperatures. This causes the nickel powder to sinter at the laser point and form a stable and loose nickel scaffold. Meanwhile, the nickel scaffolds act as templates for GFs and catalyze the carbonization of sucrose and its transformation into graphene. LIG allows direct fabrication of predesigned LIG patterns on various carbon materials, and precisely control their microstructure, conductivity, doping of heteroatoms, etc. [104]. This technology is selective and does not rely on chemicals or masks, significantly reducing the consumption of raw materials and environmental impact [105]. However, LIG technology still faces some challenges in research and application promotion, mainly including how to accurately manipulate the internal microstructure and atomic arrangement of LIG, and how to effectively apply it to more fields. In addition, the non-uniformity of energy absorption may also lead to some side reactions, which require further research and resolution.

## 5. Role of Pore Structure Regulation in Energy Applications

With the rapid development of renewable energy sources, the demand for efficient energy storage devices is growing. Supercapacitors, as a new type of energy storage device, have attracted widespread attention due to their fast charging and discharging, long lifespan, and high-power density. BDCs, as a new type of carbon material, have shown great potential in the application of supercapacitor electrode materials due to their unique porous structure and excellent conductivity.

BDC is a material prepared from biomass resources through thermochemical methods. Its hierarchical porous structure endows it with a large specific surface area, high porosity, rich pore structure, and excellent conductivity. These characteristics have a significant impact on the ion transport, specific capacitance, and charging and discharging performance of supercapacitors. The effects of different pore sizes on the charge storage and electrolyte ion diffusion of capacitors vary, and the synergistic action of micropores, mesopores, and macropores is key to improving the performance of supercapacitors.

**Figure 9:**
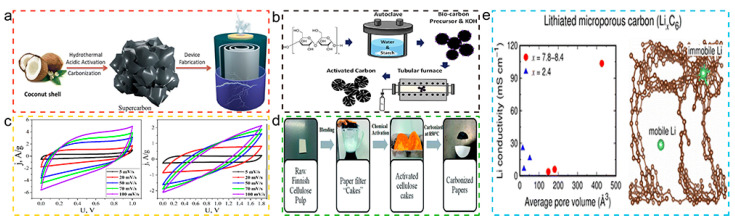
(**a**) Schematic illustration of the preparation of activated carbon for supercapacitor [106]. Copyright 2024, Royal Society of Chemistry. (**b**) Schematic of the preparation of activated biocarbon [108]. Copyright 2024, Maney Publishing. (**c**) Cyclic voltammograms of supercapacitors made from bio-carbon material NCNM with electrolyte 30% KOH and 1 M Na_2_SO_4_ [109]. Copyright 2024, Multidisciplinary Digital Publishing Institute. (**d**) Porous Characteristics of Microporous Carbon and Structure of Litigated Microporous Carbon [110]. Copyright 2018, Royal Society of Chemistry. (**e**) Schematic illustration of the synthetic procedure of activation and carbonization of precursor cellulose materials [111]. Copyright 2022, Royal Society of Chemistry.

Mostafa et al. [106], according to the steps in Figure 9a, used coconut shells as precursors to prepare two activated carbon samples: SUSCAP-01 (high micropore ratio) and SUSCAP-02 (high mesopore ratio) through carbonization and activation processes. These samples demonstrated a hierarchical porous structure of micropores, mesopores, and macropores, where micropores increased the specific surface area and served as adsorption sites for electrolyte ions, mesopores facilitated rapid ion diffusion, and macropores acted as ion buffers, reducing the ion diffusion distance and effectively enhancing the specific capacitance of supercapacitors.

BDC, with its high SSAs, abundant pore structure, and rich surface functional groups, has become a preferred material for supercapacitor electrodes. Researchers, such as Ravi et al. [107], have utilized spruce bark and activation agents, like KOH and ZnCl_2,_ to prepare efficient carbon electrodes through a one-step pyrolysis method. The pore structure is crucial for charge storage, as it affects the internal resistance of the electrode and the diffusion conditions of the charge. Biochar activated with KOH has higher SSAs, while biochar activated with ZnCl_2_ has more mesopores, which help reduce resistance and enhance the phase angle, thereby strengthening charge storage capacity. ZnCl_2_-activated biochar exhibits lower internal resistance and a higher phase angle due to its high mesopore ratio and residual Zn, which is beneficial for charge storage. The pore structure also provides transmission channels for electrolyte ions, promoting charge separation. KOH-activated biochar has a rough and porous surface, while ZnCl_2_-activated biochar features denser cavities and pores, facilitating electrolyte penetration. The ZnCl_2_-activated biochar electrode demonstrates superior supercapacitor performance, with an area capacitance as high as 342 mF/cm^2^, thanks to its hydrophilic interactions and electrolyte penetration. In summary, the pore structure plays a decisive role in charge storage for supercapacitors, affecting internal resistance, charge accumulation, and the transmission efficiency of electrolyte ions. By optimizing the pore structure, the performance of supercapacitors can be significantly improved, expanding their application prospects in energy storage.

Kang et al. [108] further confirmed the impact of pore structure on the charging and discharging rate of supercapacitors. By following the steps in Figure 9b, starch was prepared into BDCs and, by adjusting the pore structure of the starch-derived BDCs, especially the development of mesopores and macropores, the diffusion of electrolyte ions can be significantly affected, thereby enhancing the charging and discharging rate of supercapacitors. The HT-900 sample demonstrated the smallest charge transfer resistance and excellent charging and discharging performance due to its larger pore size and high degree of graphitization.

The role of micropores in supercapacitors should not be overlooked. Ewelina et al. [109] prepared nitrogen-containing nano porous carbon materials (NCNMs) through direct pyrolysis, which showed a high degree of chemical composition uniformity and developed micropore–mesopore structure. According to the data in Figure 9c, this structure helps to improve the energy performance of supercapacitors, mainly due to its large internal surface area and more active sites.

As a new type of carbon material, BDC is gradually replacing traditional battery cathode materials due to its advantages, such as high specific surface area and porous structure, chemical stability, wide range of raw materials, and preparation methods. Lee et al. [110] prepared BDCs according to the steps in Figure 9d and, by adjusting the pore structure of microporous carbon, especially the pore size and porosity, the storage capacity and ion transport rate of lithium-ion batteries can be significantly improved.

Daniel et al. [111] further confirmed the importance of optimizing the pore structure and chemical composition to improve the electrochemical stability and reversible capacity of supercapacitor electrode materials. The porous carbon material prepared by KOH activation of cellulose has a high specific surface area and total porosity. According to the data in Figure 9e, the best electrochemical performance was obtained by optimizing the KOH/cellulose ratio.

In summary, the hierarchical porous structure and excellent conductivity of BDC give it great potential in the application of supercapacitor electrode materials. By optimizing the pore structure, especially with the development of appropriately sized mesopores and macropores, the diffusion rate of electrolyte ions can be significantly improved, thereby enhancing the charging and discharging performance of supercapacitors. In addition, the chemical stability and wide range of raw materials for BDCs make them sustainable and environmentally friendly supercapacitor electrode materials. Future research should continue to explore the preparation methods and optimization of pore structures of BDCs to achieve more efficient and stable supercapacitor electrode materials.

## 6. Role of Pore Structure Regulation in Catalysis Applications

BDC exhibits a broad potential for applications in the field of catalysis due to its unique porous structure and chemical properties [112]. As a type of porous carbon material, the regulation of BDC’s pore structure is of great significance for enhancing catalytic performance. By adjusting the pore structure of BDC, its catalytic activity, selectivity, and stability can be significantly improved. The following discussion will explore the mechanisms by which the regulation of BDC’s pore structure enhances its catalytic activity, selectivity, and stability in hydrogen evolution reactions, redox reactions, and photocatalytic reactions.

The regulation of BDC’s pore structure is achieved by altering the size, distribution, shape, and number of pores. This regulation can significantly affect the catalytic activity, selectivity, and stability of BDCs. For instance, BDCs with a high specific surface area can enhance hydrogen storage capacity, thereby strengthening the catalytic role in hydrogen evolution reactions [113].

**Figure 10:**
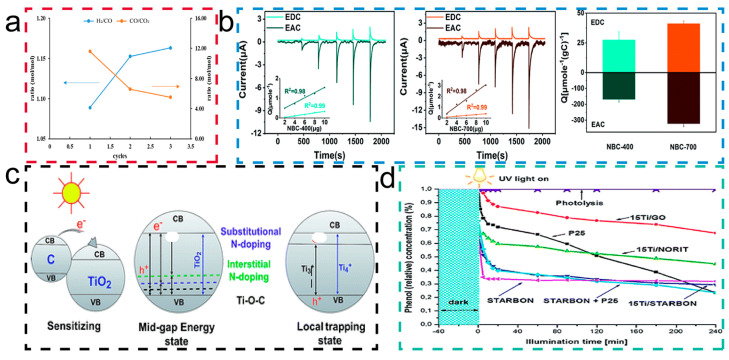
(**a**) H_2_/CO and CO/CO_2_ molar ratios based on the syngas composition versus feeding times in the combined pyrolysis and in-line catalytic reforming process using a NiBC-30 catalyst. [117]. Copyright 2023, American Chemical Society. (**b**) Reductive and oxidative current responses of NBC-400 (inset: linear relationship between the number of electrons and the added amounts of NBC-400); reductive and oxidative current responses of NBC-700 (inset: linear relationship between the electron numbers and the added amounts of NBC-700); electron transfer capacity of NBC-400 and NBC-700. [115]. Copyright 2022, Royal Society of Chemistry. (**c**) Reduction of the TiO_2_–BSP band gap energy via sensitizing, forming a mid-gap energy state and forming a local trapping state. [119] Copyright 2018, Royal Society of Chemistry. (**d**) Photocatalyst activities in the aqueous phase degradation of phenol (reaction conditions: 150 mL of mother solution, 150 mg of photocatalyst, Cphenol = 50 ppm, temperature 30 °C, reaction pressure 1 bar). [120] Copyright 2013, Royal Society of Chemistry.

Yang et al. [114] evaluated the impact of different engineered biochar-based catalysts on the production of synthesis gas during biomass pyrolysis and catalytic reforming without additional steam input. It was found that the BDC catalyst, activated physically for 2 h in Figure 10a, induced the highest H_2_/CO ratio (1.15), and activated BDCs as catalysts produced comparable yields of synthesis gas. In the studies mentioned in the article, BDCs underwent various engineering treatments, such as physical and chemical activation, nitrogen doping, and nickel doping, to enhance catalytic performance. These treatments increased BDC’s specific surface area and porosity, introduced new active sites, and thus enhanced its ability to catalyze the cracking of tar and the reforming of volatiles, improving the yield and quality of synthetic hydrogen. Therefore, a high specific surface area and hierarchical porous structure are beneficial to produce synthesis gas and hydrogen, and these characteristics of BDCs demonstrate good catalytic action in hydrogen evolution reactions.

In addition to playing a significant role in hydrogen evolution reactions, the regulation of BDCs’ pore structure also plays a certain role in redox reactions. Wu et al. [115] studied the redox properties of wheat straw-derived nanoscale biochar (NBC), particularly exploring the relationship between its pore structure and redox reactions. The typical current–time (i–t) response of NBC upon continuous addition is shown in Figure 10b. By sequentially adding different concentrations (i.e., 0.2, 0.4, 0.6, 0.8, and 1.0 mg NBC) of the sample, the electron acceptance and electron-donating reactions of NBC on the GC electrode were observed. In redox or oxidation reactions, the quantity of transferred electrons (Q) is proportional to the addition of NBC mass. As shown in Figure 10b, EAC is generally greater than EDC, indicating that most of the redox groups in BDCs are composed of oxidized groups. The results show that NBC has significant redox activity and can participate in redox reactions as an electron acceptor and donor. NBC-700, produced by high-temperature pyrolysis, exhibits higher redox activity than NBC-400 due to its higher specific surface area and porosity. This indicates that the pore structure of BDCs has a significant impact on redox characteristics. The development of the pore structure may increase the active sites on the BDC’s surface, thereby enhancing its ability to participate in redox reactions.

BDCs are often used as a catalyst, and BDC catalysts can significantly alter the distribution and properties of biomass pyrolysis products, promoting the production of bio-oil and gas through the interaction of volatiles with BDCs [116]. The relationship between the pore structure of BDCs and their performance as a catalyst in pyrolysis and catalytic reforming reactions is very close. As mentioned above, Yang et al. [117] evaluated the performance of various engineered BDC catalysts in the production of synthesis gas during biomass pyrolysis and catalytic reforming and found that the high specific surface area and hierarchical porous structure of BDCs are beneficial for the production of synthesis gas and hydrogen. Since the reactions in the study belong to thermal catalytic reactions, the pore structure of BDCs plays a role in thermal catalytic reactions. The pore structure of BDCs improves the efficiency of biomass pyrolysis and catalytic reforming processes by providing more active sites, regulating the diffusion of reactants and products, affecting the physical and chemical stability of the catalyst, and influencing the formation of coke.

The photocatalytic properties of BDCs are crucial for understanding and controlling the transformation and cycling of environmental pollutants. The photocatalytic reactions of BDCs mainly involve free radical and non-free radical redox reactions, triggered by quinone and phenolic functional groups, porous structure, and persistent free radicals in BDCs [118]. Mian et al. [119] discussed the research progress of biochar-supported photocatalysts (BSPs) and show in Figure 10c the ways to reduce the bandgap energy of titanium dioxide photocatalysts loaded on BDCs by sensitization, forming intermediate bandgap energy levels, and forming local trap states. This indicates that, by combining with BDCs, the light response range of photocatalysts can be extended to the visible light region, improving photocatalytic efficiency. From Figure 10d proposed by Colmenares et al. [120], it was observed that photocatalysts doped with different BDCs showed significant differences in the degradation efficiency of phenol, from which it can be concluded that the introduction of BDCs enhanced the performance of photocatalysts due to providing more active sites, enhancing light absorption, or promoting the separation of electron-hole pairs.

Therefore, the regulation of BDCs’ pore structure is a key means to improve their catalytic performance. By changing the pore structure, not only can the catalytic activity and selectivity of BDCs be enhanced, but also their stability and durability in various practical applications can be strengthened. Moreover, the regulation of the BDC pore structure also plays an important role in photocatalytic reactions, providing a new perspective for understanding and controlling the transformation and cycling of environmental pollutants.

Zhang et al. [121] investigated the application of sludge-derived biochar in the electrocatalytic oxidation of azo dyes (such as methyl orange), highlighting the significant impact of its porous structure on electrocatalytic performance. Firstly, the porous structure of the biochar provides more active sites, enabling effective contact with pollutants and thus significantly enhancing the efficiency of electrocatalytic reactions. Secondly, the porous structure helps to improve the mass transfer efficiency near the electrode, facilitating the exchange of materials between pollutants and the electrode, which is crucial for accelerating the rate of electrocatalytic reactions. Furthermore, as the pyrolysis temperature increases, the conductivity of the biochar is enhanced, which is closely related to the formation of the porous structure, thereby improving the transfer of electrons. Lastly, the study also points out that biochar-modified electrodes at high pyrolysis temperatures have a higher oxygen evolution potential, which can reduce side effects in the electrocatalytic process, such as the production of oxygen, thereby further enhancing electrocatalytic efficiency. In summary, the porous structure of biochar has a direct and significant impact on the enhancement of its electrocatalytic performance.

Solid waste generated by the food industry, despite its diversity, contains the potential to be transformed into a wide range of industrial application products, among which biomass-based catalysts, industrial enzymes, and biofuels are particularly prominent sustainable products. For instance, Mohd et al. [122] prepared biochar from the high-temperature calcination of green coconut waste shells, which was used in solid-state fermentation to produce microbial β-glucosidase (BGL enzyme). Its porous structure provides an abundant reaction site for the enzyme, increasing the chances of contact between the enzyme and substrate, thereby enhancing the efficiency of enzyme-catalyzed reactions. Moreover, the large specific surface area of the biochar helps to adsorb enzyme molecules, reducing their deactivation, while also promoting the transfer of substrates and products, reducing mass transfer resistance, which is crucial for improving reaction rates and efficiency. The porous structure of the biochar also facilitates the recovery of enzymes, helping to reduce costs and improve economic viability, while optimizing their catalytic performance by altering the enzyme’s microenvironment.

Furthermore, utilizing renewable resources, especially low-cost forestry waste, to produce bioenergy and materials is of great significance for achieving green and sustainable chemical processes. Xiang et al. [123] prepared biochar from sawdust through an H_3_PO_4_-assisted hydrothermal carbonization method, and then prepared a zirconium-coordinated biochar catalyst Zr-SDBC-P through a self-assembly method, which showed high activity in the catalytic transfer hydrogenation reaction of converting ethyl succinate to γ-valerolactone. Data show that the biochar treated with H_3_PO_4_ has a larger specific surface area and porous structure, which helps to improve the production efficiency of enzymes, and has good high-temperature stability, which is conducive to maintaining the stability of the catalyst during the reaction process. The recycling experiment results of the Zr-SDBC-P-4 catalyst also confirmed its good reusability, indicating that the porous structure helps to maintain the active center of the catalyst and, even after multiple uses, it can maintain its coordination catalytic performance. Therefore, the porous structure of biochar plays a key role in improving catalytic efficiency, reducing production costs, and achieving green chemical processes.

## 7. Role of Pore Structure Regulation in Environmental Applications

**Figure 11:**
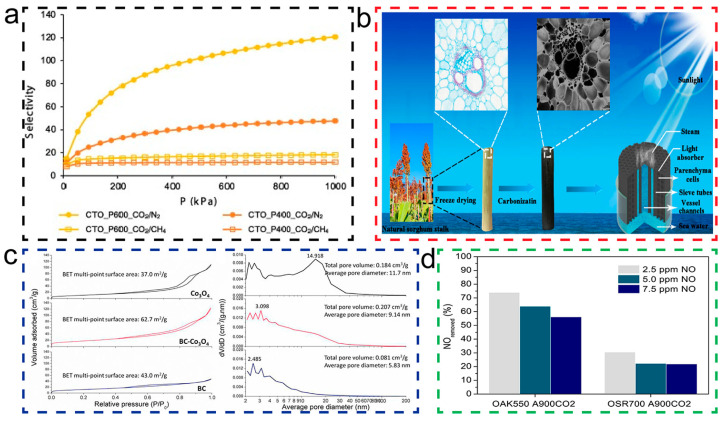
(**a**) Comparative mean selectivity, at 25 °C, of CTO_P400 (orange) and CTO_P600 (yellow), for the CO_2_/CH_4_ separation (square symbols) and for the CO_2_/N_2_, separation (circle symbols). (For interpretation of the references by color in this figure legend, the reader is referred to the web version of this article.) [124].Copyright 2023, Elsevier. (**b**) Construction and schematic process of carbonized sorghum straw for solar evaporation [126]. Copyright 2021, Multidisciplinary Digital Publishing Institute. (**c**) N_2_ adsorption/desorption isotherm and pore size distribution of the three samples [127]. Copyright 2018, Elsevier. (**d**) NO removal capacity of activated carbons as a function of the NO concentration in the air stream. Experimental conditions: Temperature: 25 °C; carbon bed: 1.5 g [129]. Copyright 2023, Elsevier.

Firstly, the pore structure of BDCs can be designed and prepared through specific processes to achieve molecular size selective separation. This size selectivity is achieved by controlling the size and distribution of pores, allowing only molecules of specific sizes to enter and be adsorbed within the pores. For instance, Mirtha et al. [124] prepared sponge-like BDC adsorbents through freeze-drying and pyrolysis, which enhance the adsorption of CO_2_ from CO_2_/CH_4_ and CO_2_/N_2_ gas mixtures. As shown in Figure 11a, by altering the pyrolysis temperature, the porosity and specific surface area of BDCs can be adjusted, providing more adsorption sites and enhancing the gas adsorption capacity. This finding indicates that, by finely tuning the pore structure of BDCs, their separation performance in gas mixtures can be significantly improved. In addition, the pyrolysis temperature is able to alter the porosity of biochar, according to Reis et al. [125] When exploring the synthesis methods of sustainable activated biochar as carbon-free anode materials for lithium-ion batteries (LIBs) and sodium-ion batteries (NIBs), researchers have found that there are significant differences in the pore structures of biochar synthesized from Norwegian spruce bark using chemical activation methods (with ZnCl_2_ and KOH). Specifically, the biochar activated by ZnCl_2_ (Biochar-1) formed highly mesoporous carbon, with 96.1% of its structure being mesopores, while the biochar activated by KOH (Biochar-2) showed a lower degree of graphitization and a more disordered, defective carbon structures, with a mesoporosity content of only 56.1%. Furthermore, Biochar-1 demonstrated the formation of more ordered graphene layers in its structure, whereas Biochar-2 exhibited a greater degree of disorder. In terms of surface functionality, although both biochars showed high functionality, Biochar-1 had a better electrochemical response due to the presence of pyridinic nitrogen functional groups. Consequently, the article concludes that, owing to its highly developed mesoporous structure and ordered carbon layer structure, Biochar-1 outperforms Biochar-2 in electrochemical properties, making it an excellent biomass anode material for LIB and NIB applications. Therefore, the article concludes that, due to Biochar-1’s highly developed mesoporous structure and ordered carbon layer structure, its electrochemical performance is superior to Biochar-2, making it an outstanding biomass anode material for LIB and NIB applications.

Secondly, the hierarchical pore structure of BDCs provides a large surface area, which increases the contact opportunities between molecules and separation materials, thereby enhancing adsorption capacity and selectivity. This high specific surface area characteristic makes BDCs an efficient adsorbent, especially when dealing with low-concentration gases. Additionally, the hierarchical pore structure also helps to reduce light reflection and enhance light scattering effects, thereby improving solar energy absorption rates, which is particularly important for solar-driven water purification technologies.

Furthermore, the hierarchical pore structure of BDCs also shows great potential in water purification applications. As shown in Figure 11b, Zhang et al. [126] used BDCs derived from sorghum stalks, prepared through a simple carbonization process, to create a solar evaporator that performed excellently in the purification of various water sources, including desalination of seawater, treatment of acidic and alkaline wastewater, and treatment of organic polluted water. The success of these applications proves that BDCs have broad application prospects not only in the field of gas separation but also in liquid separation.

In summary, the hierarchical pore structure of BDCs plays a crucial role in both gas and liquid separation fields. By finely tuning the pore structure, not only can the adsorption efficiency and selectivity be improved, but the rate and efficiency of catalytic reactions can also be enhanced. Therefore, BDCs, as a sustainable and environmentally friendly material, have broad application prospects in separation science and technology. Future research should continue to explore the relationship between the pore structure of BDCs and separation performance to develop more efficient and economical separation materials, contributing to environmental protection and sustainable development.

When discussing the role of BDCs in environmental management, the research by Chen et al. [127] provides a powerful perspective. They found, as shown in Figure 11c, that BDC modification significantly increased the specific surface area and pore structure of Co_3_O_4_, which is generally conducive to the occurrence of catalytic reactions. The biochar-modified Co_3_O_4_ catalyst showed a significant promotional effect in the heterogeneous activation process of peroxy-monosulfate (PMS). This finding not only confirms the extraordinary activity of Co_3_O_4_ synthesized with BDC assistance in PMS activation but also highlights its potential application in the efficient degradation of ofloxacin, further emphasizing the promising role of BDCs as a catalyst for the removal of organic compounds.

The research by Jin et al. [128] further deepens our understanding of the catalytic and adsorption performance of BDCs in organic compounds. They pointed out that BDCs prepared under high-temperature pyrolysis conditions (BC900) have excellent adsorption capacity for trichloroethylene (TCE) and show excellent catalytic activity in the activation of persulfate (PS) for the degradation of sulfamethazine (SMT). This finding not only confirms the adsorption and catalytic performance of BDCs in water treatment but also reveals the significant impact of BDC particle size and pyrolysis temperature on performance. It is particularly noteworthy that BC900 with a smaller particle size (0–75 μm) is 19.5–62.3% more efficient in TCE adsorption than larger BDC particles. Moreover, the removal rate of SMT using the BC900/PS system increased significantly with the decrease in BDC particle size, from 24.2% to 98.3%. This trend indicates that the particle size and pyrolysis temperature of BDCs are key factors affecting adsorption and catalytic performance in water treatment.

In summary, the particle size and pyrolysis temperature of BDCs have a significant impact on their adsorption and catalytic performance in water treatment. BDCs with smaller particle size, after high-temperature pyrolysis, show excellent TCE adsorption and SMT degradation performance due to their larger specific surface area and rich microporous structure. These findings provide new strategies for the application of BDCs in environmental management and open new directions for future research and application.

In addition to organic pollutants, in the environmental field, BDCs also play a role in the management of exhaust gases. The research by Díaz-Maroto et al. [129] provides in-depth insights into the NO removal capacity of BDCs. They found, as shown in Figure 11d, that when the amount of BDCs is halved (from 3 g to 1.5 g), the NO removal capacity of both BDCs decreases. For an initial concentration of 5 ppmv of NO, the removal capacity of both activated carbons decreased by about 36% and 40%, respectively. Further analysis indicates that the specific surface area (SBET) of BDCs is directly related to NO removal capacity. A larger specific surface area means more active sites, which not only helps in the adsorption of NO molecules but also promotes the subsequent oxidation process. Moreover, the total pore volume and pore size distribution of BDCs are also important factors affecting NO removal capacity. BDCs with a larger micropore volume can provide more contact opportunities for NO and O_2_, thus promoting the oxidation reaction. The role of BDC pore structure in exhaust gas treatment cannot be ignored, as it directly affects the adsorption of NO molecules, contact with oxygen, and subsequent catalytic oxidation reactions. These findings emphasize the potential of BDCs’ pore characteristics and surface chemical properties in improving their performance in practical exhaust gas treatment applications. By optimizing these characteristics, we can expect significant improvements in the performance of BDCs in the field of exhaust gas treatment.

BDCs, as a carbonaceous material derived from biomass pyrolysis, play an important role in the treatment and recovery of heavy metals. Cao et al. [130] developed a new type of BDC by using dehydrated algal waste and combining it with KOH and FeCl_3_ for co-activation treatment, preparing a highly efficient adsorbent specifically for elemental mercury in coal combustion flue gas. This method of preparing BDCs not only achieves the resource utilization of waste but also significantly increases the material’s specific surface area and porosity, thereby enhancing its adsorption capacity for mercury. Algal waste-derived BDCs show excellent performance in removing elemental mercury, far exceeding that of unmodified BDCs. This high removal capacity is attributed to the Fe and Cl elements on the surface of the BDCs, which are retained during the KOH activation process and enhance the BDCs’ magnetic properties by forming Fe-Cl complexes, thus simplifying the recovery process of the adsorbent. In the management of heavy metal pollution, the application of BDCs is mainly based on their high specific surface area and abundant surface functional groups, which enable BDCs to provide many active sites to effectively adsorb heavy metal ions in the solution, such as lead (Pb), cadmium (Cd), mercury (Hg), and chromium (Cr), etc. [131]. In summary, the application prospect of BDCs in heavy metal pollution management is broad, and their low cost, environmentally friendly, and adjustable characteristics make them an effective tool for heavy metal removal. With in-depth research, BDCs are expected to play a more significant role in the management and control of heavy metal pollution.

In summary, we have compiled a table (Table 1) to illustrate the adsorption efficiency of various biochars for different environmental pollutants based on the readings of some articles and the adsorption of pollutants by biochars in the environment.

**Table 1:** The adsorption efficiency of various biochars for different pollutants in the environment.

Types of Biochar	Adsorbed Contaminants	The Size of the Contaminant	Separation Efficiency	Reference
Carbonized sorghum straw	Seawater, acidic/alkaline wastewater, organic wastewater		Energy conversion efficiency 100%, Evaporation rate 3.173 kg m^−2^ h^−1^	[126]
Coconut shell activated carbon	Oils and organic solvents		The oil–water separation efficiency is maintained at more than 98%.	[132]
Biochar residue/ethylcellulose mixed matrix membrane after lignin depolymerization	CO_2_/CH_4_ and CO_2_/N_2_	Molecular level	CO_2_/CH_4_’s selectivity is 9.97, CO_2_/N_2_’s electivity is 20.01	[133]
Magnetic biochar (M-RSB and M-SSB)	Cd^2+^ in solution	Ionic level	The maximum adsorption capacities are as follows: 42.48 mg/g (M-RSB) and 4.64 mg/g (M-SSB)	[134]
Biochar derived from wheat straw (SB)	Cd^2+^ and Co^2+^ in solution	Ionic level	The maximum adsorption capacities are as follows: 193 μmol g^−1^ (Cd^2+^) and 89.7 μmol g^−1^ (Co^2+^)	[135]
Pine needles magnetic biochar (pncm and pncom)	Cu (II)	Ionic level	pncm: 0.4 mmol/g, pncom: 1.0 mmol/g	[136]
Peanut Shell Biochar (PSB) and Modified PSB (MPSB)	As (III) and As (V)	Ionic level	MPSB: As (III) 86, As (V) 91.26%	[137]
Distiller’s grains are derived from biochar	Pb^2+^	Ionic level	79.12 mg/g	[138]
Crab shell-derived biochar (CSAB)	Diesel Oil		93.9 mg/g	[139]
Sheep Manure Biochar (SMB) and Robinia Biochar (RPB)	Mixed heavy metals (Pb^2+^, Cu^2+^, Cd^2+^)	Ionic level	SMB3: Pb^2+^ 20.2 mg/g, Cu^2+^ 13.9 mg/g, Cd^2+^ 3.2 mg/g RPB8: Pb^2+^ 7.4 mg/g, Cu^2+^ 10.5 mg/g	[140]

## 8. Conclusions and Outlook

Pore engineering in BDCs has garnered increasing attention due to its significant impact on enhancing the performance of these materials across a wide range of applications, such as energy storage, catalysis, and environmental remediation. The ability to tailor the pore structure, including pore size, distribution, and interconnectivity, allows for the optimization of specific physical and chemical properties that directly influence the material’s efficiency and adaptability in diverse technological applications.

In energy storage, the hierarchical porous structure of BDCs has shown immense potential in improving the performance of devices like supercapacitors, lithium-ion batteries, and sodium-ion batteries. The combination of micropores, mesopores, and macropores within BDCs enhances both ion transport and charge storage capabilities. Micropores offer a high surface area for ion adsorption, which increases the energy storage density, while mesopores and macropores reduce ion diffusion pathways and enhance electrolyte transport, improving power density and charge–discharge rates. As such, optimizing the pore architecture is critical for achieving high-performance energy storage devices. Furthermore, the tunability of pore size distribution in BDCs is essential for balancing energy density with power density in energy storage applications.

In catalysis, pore-engineered BDCs are highly effective due to the increased accessibility of active sites and the improvement of mass transport. The hierarchical porous structures facilitate the diffusion of reactants and products, which is especially critical in heterogeneous catalysis where the interplay between surface area and pore architecture determines the catalytic efficiency. The ability to create a finely tuned pore network not only boosts catalytic activity but also enhances selectivity and stability, particularly in reactions such as hydrogen evolution, oxygen reduction, and carbon dioxide reduction. BDCs with well-defined pore structures are increasingly being used as metal-free catalysts or as support for single-atom or nanoparticle catalysts, owing to their high specific surface area and chemical stability.

Environmental applications of BDCs, particularly in gas separation, water purification, and pollutant removal, benefit significantly from precise pore structure control. The hierarchical porosity of BDCs facilitates the adsorption of gases, such as CO_2_, NOx, and volatile organic compounds (VOCs), through molecular sieve effects and enhanced surface interactions. In water treatment, the combination of micro- and mesopores enables efficient adsorption of organic and inorganic pollutants, while macropores improve fluid flow and regeneration capacity. The adaptability of BDCs in environmental remediation is further expanded by chemical modifications, such as doping with heteroatoms, which improve their affinity for specific contaminants and enhance photocatalytic or redox capabilities. Moreover, recent advancements in pore engineering have enabled the design of BDCs with tunable surface chemistry, further expanding their applicability in environmental cleanup.

Looking forward, further advancements in pore engineering techniques are essential to unlock the full potential of BDCs. Novel strategies such as dual-template synthesis, self-activation methods, and advanced templating techniques, which combine both hard and soft templates, are promising pathways to achieve precise control over pore architectures and enable the development of hierarchical structures with optimized performance. The integration of these strategies with emerging carbonization techniques, such as microwave and laser carbonization, presents new opportunities to fine-tune the pore structure at the nanoscale, leading to enhanced performance in energy, catalysis, and environmental applications.

Moreover, the combination of BDCs with other functional materials, such as metal oxides, metal-organic frameworks (MOFs), and conductive polymers, can create hybrid materials with synergistic properties, further expanding their functionality. The development of BDCs-based composites or heterostructures could lead to breakthroughs in applications such as electrochemical catalysis, gas sensing, and energy harvesting. For instance, incorporating transition metals into the porous carbon framework can generate active sites for electrocatalysis, while maintaining the high conductivity and structural integrity of the carbon matrix.

In conclusion, pore engineering is a pivotal strategy for maximizing the performance of BDCs. By refining control over pore structure, enhancing surface chemistry, and exploring new synthesis routes, BDCs are poised to play an increasingly critical role in addressing global challenges related to renewable energy, environmental sustainability, and green technology. Future research should focus on bridging the gap between laboratory-scale synthesis and large-scale production of BDCs, ensuring that these materials can be manufactured in an economically viable and environmentally sustainable manner. Additionally, further exploration of the relationships between pore structure, surface functionality, and material performance will be key to developing next-generation BDCs with tailored properties for specific applications. By continuing to innovate in pore engineering, BDCs can become a cornerstone of future technologies that contribute to a sustainable and circular economy.
